# The effect of virtual reality on skin prick test-related pain and fear in children: a randomized controlled trial

**DOI:** 10.1007/s00431-025-06262-x

**Published:** 2025-06-18

**Authors:** Cihangir Sahin, Sule Cinakli, Simge Atar Bese, Gulten Tuncerler, Nur Torer, Duygu Erge, Pinar Uysal

**Affiliations:** Department of Pediatric Allergy and Immunology, School of Medicine, Aydın Adnan Menderes University, (ADU Hospital, E Block, Room Number:42), 09100 Efeler, Aydın, Turkey

**Keywords:** Allergy, Children, Clinical trial, Fear, Pain, Pediatrics, Skin prick test, Virtual reality

## Abstract

Skin prick tests are a common method for diagnosing allergies in pediatric allergy clinics. Unfortunately, these tests can be distressing for children, causing pain and fear that can make them challenging to conduct. Virtual reality (VR) can be used to distract children during medical procedures. This study examines the effect of VR on skin prick test-related pain and fear in children. This parallel-group, single-blind, prospective, randomized controlled trial enrolled 233 children evaluated at the Pediatric Allergy Clinic and scheduled to undergo skin prick testing between March and June 2024. Among these children, 75 children aged 4–10 years who met the inclusion criteria were divided into two groups as VR group (*n* = 25) and control group (*n* = 25) by block randomization using gender and age group variables. The VR group wore VR goggles during the skin prick test, while the control group underwent the standard procedure. Pain and fear levels were assessed using the Wong-Baker FACES® Pain Scale and the Child Fear Scale. Children in the VR group exhibited a significant reduction in pain and fear levels associated with the skin prick test experience compared to the control group, as evaluated by children, parents, and researchers (*p* < 0.05). No side effects related to VR were reported.

*Conclusion*: Virtual reality can be an effective tool in pediatric allergy clinics to reduce pain and fear during skin prick testing. By enhancing the child’s comfort, VR may also improve adherence to allergy follow-up and treatment, ultimately contributing to better clinical outcomes.

**Table:** 

**What is Known:** *• Children often experience significant pain and fear during invasive procedures such as skin prick tests, and distraction methods have been shown to reduce pain and anxiety.* **What is New:** *• This study is the first randomized controlled trial to demonstrate that virtual reality (VR) significantly reduces pain and fear during skin prick tests in children.* *• This study supports the integration of new technologies such as VR as a standard practice for managing pain and fear during skin prick tests in pediatric allergy clinics.*

*Trial registration*: The clinical trial registration number is NCT06421779. Study start date: March 22, 2024. Study completion date: July 22, 2024. (https://clinicaltrials.gov/study/NCT06421779).

**Graphical Abstract:**

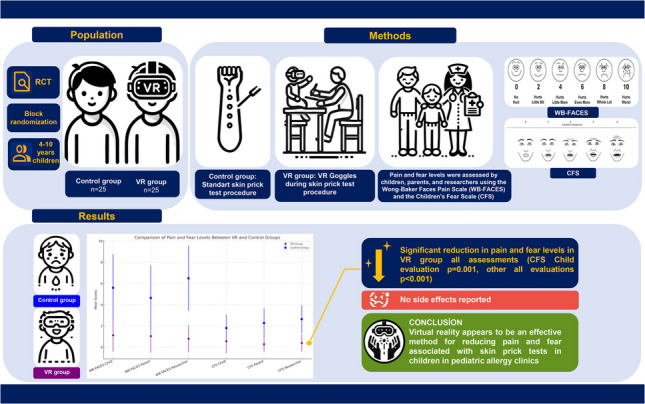

## Introduction

Children are exposed to numerous invasive procedures, such as blood collection and vascular access, that cause pain and fear during the diagnostic and therapeutic processes [1, 2]. Since pain and fear result in several physical, cognitive, and psychological effects, their management is of great importance, particularly in children [3]. If this process is poorly managed, children may come to regard hospitals as places of pain and fear. Pain and fear during interventional procedures in pediatric patients are among the most difficult problems to manage in hospitals [4]. Approximately 50–60% of children experience pain and fear during invasive procedures, and 63% of these develop a needle phobia [5–7]. Unpleasant medical experiences in childhood, particularly those involving needles, can lead to increased sensitivity to pain, needle phobias, medical trauma with pretreatment anxiety disorders, and treatment resistance as a consequence; as well as an increased risk of anxiety and avoiding care in adulthood [8–11].

The skin prick test is the gold standard diagnostic method most frequently used in pediatric allergy clinics for detecting allergens with high levels of sensitivity, specificity, and reliability [12, 13]. The numerous pricking procedures performed during the skin test may develop into a painful and frightening experience for children.

Skin prick tests present unique challenges for children as they involve multiple needle pricks performed on the visible forearm area, allowing children to see each prick as it occurs. Unlike single needle procedures, skin prick tests typically require approximately 20 separate pricks with standardized allergen extracts. Additionally, children may experience itching sensations during the 15–20 min observation period, which can be distressing and may cause them to want to scratch the test sites, potentially affecting results [14].

The distraction method is a commonly used technique for reducing pain and fear during interventional procedures [15–18]. Effective distraction should be engaging and immersive, stimulating as many senses as possible. Clinical studies show that combining visual and auditory distracting stimuli is more effective than using visual stimuli alone [18]. Virtual reality (VR) offers these qualities as an advanced technology that creates realistic simulations in which users can explore and interact with different worlds and experiences [19–21]. VR is typically applied using a headset or goggles that can create a virtual world distinct from children’s current physical environment, effectively distracting them from the potential experience of pain and fear.

Few studies have examined the impact of VR on pain and fear during hospital procedures. Most focus on procedures for children, such as blood draws, injections, burn treatments, or dental care, while a smaller number highlight VR’s benefits in adult procedures. Recent studies, systematic reviews, and meta-analyses have reported that VR reduces pain and fear associated with invasive procedures [19–27].

While previous studies have demonstrated VR’s effectiveness for single needle procedures, skin prick tests present unique challenges that make them particularly suitable for VR intervention: (1) multiple needle pricks increase cumulative pain and anxiety, (2) the procedure occurs in the child’s direct line of sight, making distraction more critical, (3) the 15–20 min observation period provides sufficient time for meaningful VR engagement, and (4) unlike procedures requiring precise positioning, children can comfortably wear VR goggles throughout skin prick testing without interfering with the procedure.

This is the first study that aims to show the effect of VR on skin prick test-related pain and fear in children.

## Methods

### Study design

This parallel-group, single-blind, prospective, randomized controlled trial screened children evaluated at the skin testing unit of a tertiary pediatric allergy referral center in Türkiye between March and June 2024 (ClinicalTrials.gov registration NCT06421779). The study adhered to the CONSORT guidelines [28].

### Sample size and randomization

The number of participants was determined using data obtained from the reference study by Erdoğan et al. [19]. Power analysis was performed based on Wong-Baker FACES® Pain Rating scale data from the reference study comparing the VR group with the control group, which used the same scale as our primary outcome measure. At calculation by G*Power 3.1.9.7, the effect size was 0.92, the alpha margin of error was 0.05, and the statistical power was 80%, and it was determined that at least 19 participants in each group would be required. The analysis showed that a total sample size of 38 participants would be sufficient to detect significant differences. In consideration of potential losses of 10% that might occur during the study, we planned to include at least 21 participants in both groups for a total of 42 participants.

Previous studies have shown that gender and age affect pain and fear associated with invasive procedures in children [29]. Seventy-five children aged 4–10 years who met the inclusion criteria were divided into two groups as the VR group (*n* = 25) and the control group (*n* = 25) by block randomization using gender (boys and girls) and age group variables (4–6 years/preschool and 7–10 years/school). A blocked randomization list was developed using an online randomization tool [30]. This age group was selected since the Wong-Baker FACES® Pain Rating Scale (WB-FACES®) and the Children’s Fear Scale (CFS), which we use for pain and fear assessment, have a common validation age range for Turkish children.

### Study population

The criteria for inclusion in the study were (1) age 4–10 years and (2) the skin prick test being required due to allergic diseases, and (3) being scheduled to undergo a skin prick test an aeroallergen panel.

### Exclusion criteria

Exclusion criteria were (1) the presence of chronic diseases (except for allergic diseases), (2) vision, hearing, and speech disorders, (3) psychiatric and neurological diseases (including epilepsy, cerebral palsy, developmental delays, autism spectrum disorders, any condition affecting cognitive function or ability to cooperate with procedures), (4) mental status, learning and perception disorders, (5) a history of pre-procedure sedative, analgesic or narcotic drug use within the previous 24 h, (6) a history of active infection, (7) being scheduled to undergo a skin prick test other than an aeroallergen panel, (8) contraindications for skin prick testing, and (9) recurrent skin prick test history.

### Ethics

This study was approved by the Clinical Research Ethics Committee of Aydın Adnan Menderes University (Approval Number: 2024/66) in accordance with the “Declaration of Helsinki.” All participants took part voluntarily, personal written informed consent being obtained from the parents of all children, and verbal consent from the children before participation.

### Data collection tools

Data were collected by means of a case report form created using WB-FACES® and CFS. Both scales are suitable for personal, parental, and researcher evaluation [31, 32]. In the present study, children’s personal, parental, and researcher reporting data were evaluated for both scales.

### Wong-Baker FACES® Pain Rating Scale (WB-FACES®)

**Fig. 1:**
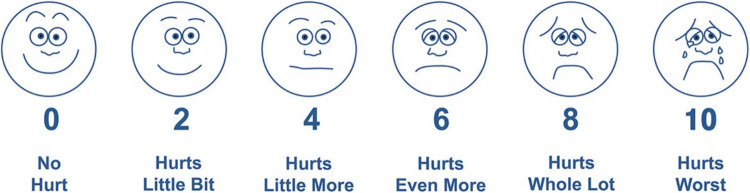
The Wong-Baker FACES® Pain Rating Scale

WB-FACES® was developed by Wong and Baker in 1981 and revised in 1983. WB-FACES® is a reliable scale that has been validated for the Turkish population [33–35]. The scale is used to diagnose pain in children aged 3–18 years. It consists of six facial expressions, each representing an increasing degree of pain scored from 0 to 10 from left to right (0 = very happy face/no pain, 10 = a crying face/worst pain imaginable) (Fig. 1) [31].

#### Children’s Fear Scale (CFS)

**Fig. 2:**
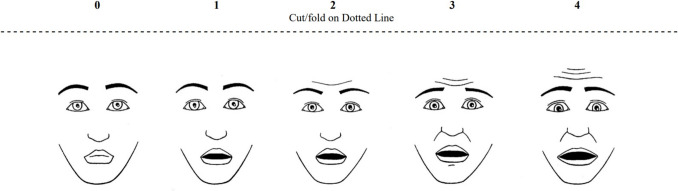
The Children’s Fear Scale

The CFS was developed by McMurtry et al. in 2011 [32]. It was adapted into Turkish by Gerçeker et al. in 2018 in the context of children aged 4–10 years [36]. It consists of five facial expressions representing a range from neutral (0) to extreme fear (4) (Fig. 2).

### Virtual Reality (VR)

Virtual reality represents an advanced technology that offers a 360-degree visual and audio simulation that surrounds the user and allows him to look in all directions [37]. In this study, the VR procedure was performed using a smartphone (Samsung Galaxy S23 Ultra), a VR goggle (Schulzz VRG Pro), and a VR underwater experience video (2160p resolution video suitable for VR format) capable of attracting the attention of the age groups involved. A small, open-design VR goggle that does not cover the forehead and in which the phone is placed externally was preferred to facilitate the assessment of the scales during the procedure.

### Procedure

All children were admitted to the skin testing unit with their parents. The researcher informed the participants and parents about the skin prick test and scales (WB-FACES® and CFS). Before the procedure, all participants were asked verbally about their baseline fear status concerning the skin prick test. Standard care in our clinic typically includes parental presence, verbal reassurance from healthcare providers, and allowing children to bring a comfort item. No formal distraction techniques such as toys, videos, or music were routinely used. For this study, participants were specifically instructed not to use any additional distraction methods to ensure the intervention effect could be properly evaluated.

#### VR equipment and setup

We deliberately selected an open-design VR goggle (Schulzz VRG Pro) that positions the smartphone externally to minimize coverage of facial areas. Headset straps were carefully adjusted for each patient to avoid covering the forehead area. Virtual reality goggles were sterilized after each participant’s use.

#### Intervention

**Fig. 3:**
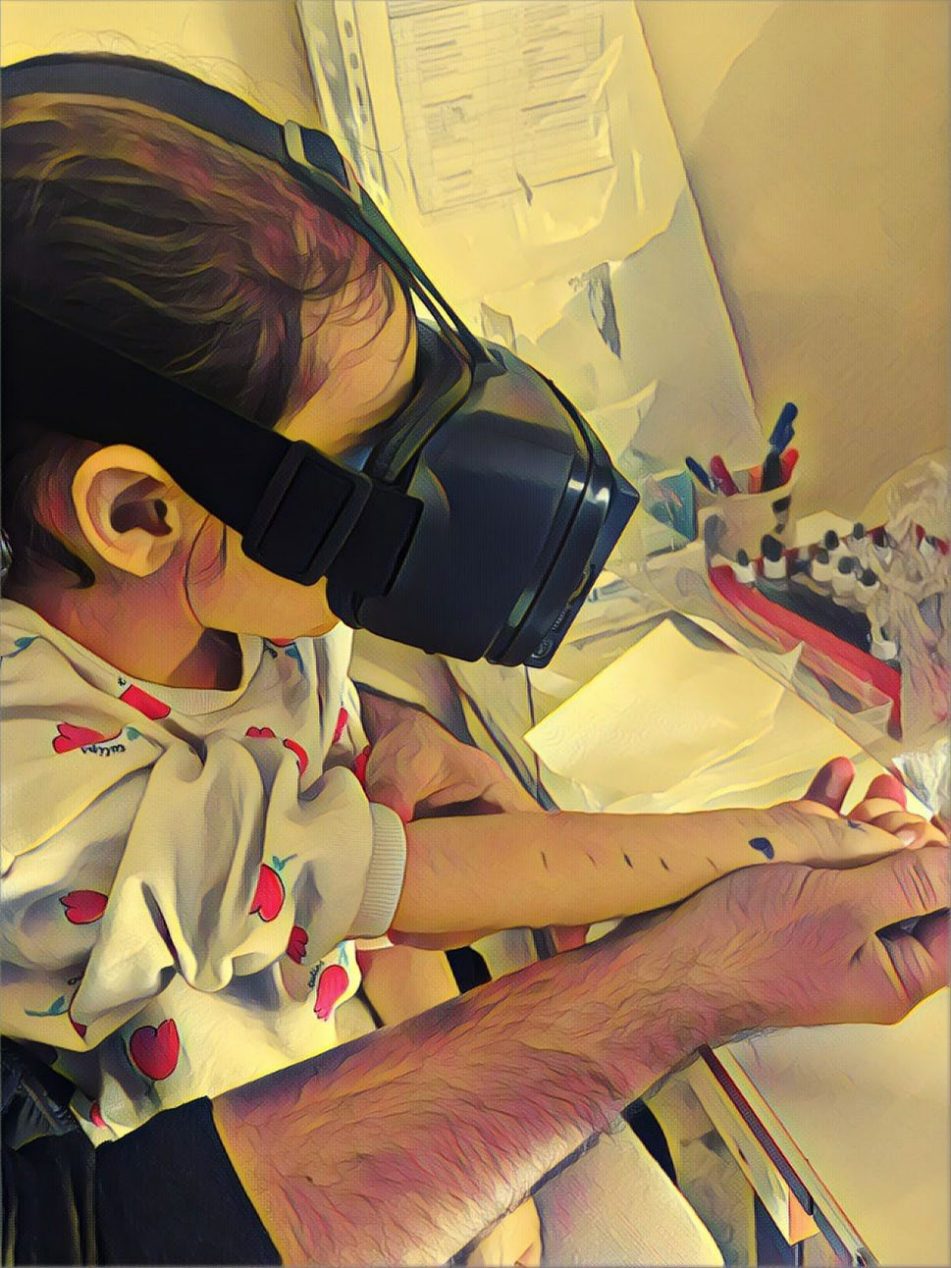
Illustration of VR-assisted skin prick testing

Participants in the VR group wore VR goggles for approximately 2 min before the skin prick test and watched a VR underwater experience video throughout the procedure (Fig. 3). The control group underwent the routine skin prick test procedure without any distraction intervention.

#### Assessment protocol

To enable comprehensive pain and fear evaluation, we implemented a multi-faceted assessment approach: (1) behavioral observations of visible indicators such as body tension, movement, and verbal expressions during the procedure, (2) formal pain and fear scale assessments conducted immediately after the procedure, and (3) evaluation by three different assessors (child, parent, and trained observer nurse) to capture multiple perspectives and minimize assessment bias.

#### Data collection

All assessments occurred immediately after completion of the skin prick test (before the 15–20 min observation period for reading results). To minimize observer bias, a different specialist nurse (not the one who performed the procedure) who was specifically trained in the use of WB-FACES® and CFS scales conducted the pain and fear assessments. This observer nurse had prior experience in pediatric pain and fear assessment and received additional training on proper scale administration before the study. For children in the VR group, scale assessments were conducted directly after removing the VR goggles while the experience was still fresh in their memory. The evaluation sequence was standardized: first, the trained observer nurse asked the child to indicate their pain and fear levels, then asked the parent to provide their assessment, and finally completed her own evaluation. All assessments were recorded independently before any discussion between evaluators.

### Skin prick test

Skin prick tests were performed over the same period by a nurse with at least 5 years’ relevant experience. All patients underwent the same aeroallergen skin test panel. The tests were performed using a prick test applicator (MedBlue One, Türkiye) on the flexural aspect of the forearm by standard guidelines using standardized glycerinated extracts (1% weight/volume) from LOFARMA (Milan, Italy).

### Statistical analysis

SPSS version 23.0 statistical software (IBM SPSS Inc., Chicago, IL, USA) was used for statistical analysis. Categorical variables were presented as numbers (%), and continuous variables as mean ± SD and median values (interquartile range (IQR)). Univariate analyses including categorical data were performed using the *χ*2 test. Data normality was assessed using the Shapiro–Wilk normality test, after which parametric values were analyzed using the Student’s *t*-test and non-parametric variables using the Mann–Whitney *U* test. A *p*-value of < 0.05 was considered statistically significant.

## Results

**Fig. 4:**
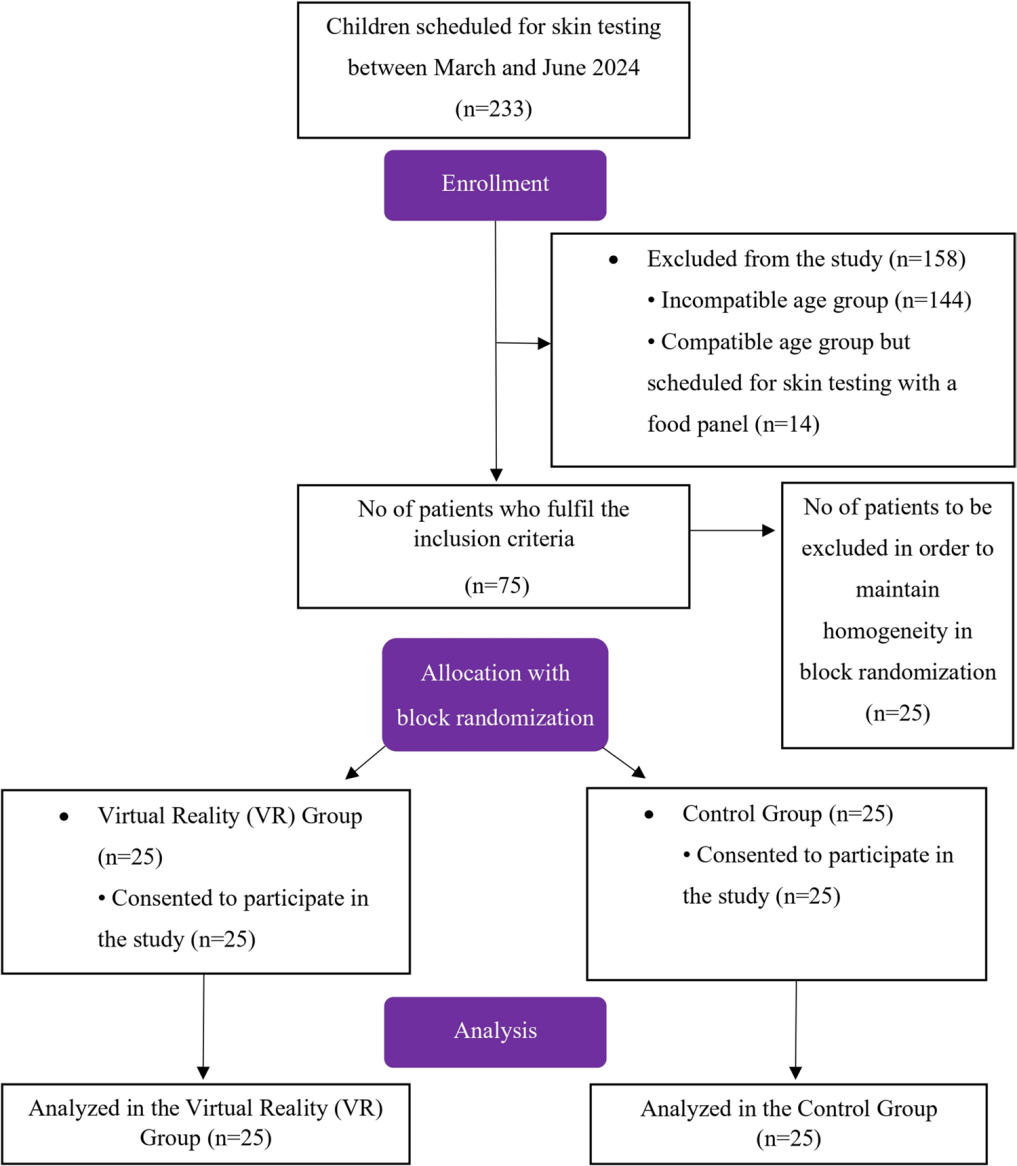
Study flow chart

Two hundred thirty-three children evaluated at the pediatric allergy clinic and scheduled for skin prick testing between the study period were screened for the study. There were 75 children aged between 4 and 10 years old who met the inclusion criteria; however, only 50 of them were allocated either to VR (*n* = 25) or control (*n* = 25) groups in order to maintain homogeneity recommended by the online block randomization tool. The study flowchart is shown in Fig. 4.

No significant differences were found between the VR and control groups in terms of demographic data such as age, age groups, and gender, or of the frequency of basal fear before the skin prick test (*p* > 0.05) (Table 1). Side effects were assessed through a systematic evaluation process that included post-procedure observation during the 15–20 min waiting period, a detailed follow-up examination, and direct questioning of participants using a standardized checklist including headache, dizziness, nausea, eye strain, and discomfort. No side effects related to VR were reported by participants in the VR group. Information about the novelty of the VR experience was obtained through brief informal interviews with children and parents immediately after the procedure, where they were asked about their previous experience with VR technology. All children in the virtual reality group and their families said that VR goggles were a new and novel experience for them.

**Table 1:** A comparison of demographic characteristics and frequency of basal fear before the Skin Prick Test between the virtual reality and control groups

	Virtual reality (VR) (*n* = 25)	Control (*n* = 25)	*p*-value (< 0.05)*
Age (years) (mean ± SD^a^)	6.72 ± 1.51	6.64 ± 2.03	0.876^†^
Age group, *n* (%)			
4–6 years (preschool age)	13 (52%)	14 (56%)	0.777^‡^
7–10 years (school age)	12 (48%)	11 (44%)	
Gender, *n* (%)			
Male	13 (52%)	12 (48%)	0.777^‡^
Female	12 (48%)	13 (52%)	
Frequency of basal fear before test (%)	48%	40%	0.569^‡^

*P <.05 is significant

^a^Mean ± standard deviation

^†^Comparison of normally distributed continuous variables was performed using the Student’s *t*-test

^‡^Comparison of categorical variables was performed using the *χ*2 test

Pain levels associated with the skin prick test in the VR group were lower than those in the control group at evaluations performed by children, parents, and researchers using the WB-FACES® scale (*p* < 0.001 for all) (Table 2).

**Table 2:** A comparison of pain and fear levels between the virtual reality and control groups using the Wong-Baker FACES® Pain Rating Scale and Children’s Fear Scale

	Virtual reality (VR) group (*n* = 25)	Control group (*n* = 25)	*p*-value (*p* < 0.05)	Effect size (Cohen’s *d*)
Pain assessment				
WB-FACES® Child Evaluation (mean ± SD)	1.12 ± 1.53	5.60 ± 3.16	** < 0.001**	1.80
WB-FACES® Parent Evaluation (mean ± SD)	1.04 ± 1.54	4.64 ± 3.09	** < 0.001**	1.47
WB-FACES® Researcher Evaluation (mean ± SD)	0.80 ± 1.29	6.48 ± 3.07	** < 0.001**	2.41
Fear assessment				
CFS Child Evaluation (mean ± SD)	0.56 ± 1.15	1.80 ± 1.29	**0.001**	1.01
CFS Parent Evaluation (mean ± SD)	0.28 ± 0.73	2.28 ± 1.36	** < 0.001**	1.83
CFS Researcher Evaluation (mean ± SD)	0.40 ± 0.81	2.64 ± 1.31	** < 0.001**	2.05

Parametric data analysis was done by using the Student’s *t*-test

*p*-values in boldface mark statistical significance

WB-FACES®: Wong-Baker FACES® Pain Scale, CFS: Children’s Fear Scale

Fear levels associated with the skin prick test were also lower in the VR group than in the control group at evaluations by children, parents, and researchers using the CFS scale (*p* = 0.001, *p* < 0.001, and *p* < 0.001, respectively) (Table 2).

## Discussion

This study is the first in the literature to investigate the effect of VR on pain and fear associated with skin prick testing in children. Pain and fear levels were assessed by three different evaluators (the child, a parent, and a researcher). The VR group exhibited lower levels of pain and fear at all assessments compared to the control group. The results of this study suggest that VR is effective in reducing pain and fear associated with skin prick testing.

Virtual reality has previously been reported to significantly reduce procedural pain in children [6, 25, 33, 38]. In a randomized controlled trial, Gerçeker et al. reported lower pain levels in children aged 5–12 years who received VR interventions compared to a control group during blood draw [33]. Another study described VR as an effective and safe method for reducing pain in children aged 4–11 years undergoing intravenous insertion and blood draw procedures [6]. Similarly, systematic reviews and meta-analyses reported significant reductions in pain levels when VR was used as a distraction tool during various medical procedures [25, 38].

When considering pain management strategies for skin prick testing, it is important to acknowledge that traditional pharmacological approaches have limitations [39, 40]. While topical anesthetics could theoretically reduce procedural pain, they are generally not recommended for routine use in skin prick testing due to several important constraints [14, 41]. Topical anesthetics like EMLA cream (lidocaine/prilocaine) can suppress the erythema (redness) component of allergic reactions without consistently affecting wheal size, potentially complicating test interpretation [39]. Additionally, optimal effect requires 2–3 h pre-application, making them impractical for routine clinical use [40, 41]. Other concerns include potential allergic contact dermatitis to anesthetic components, local skin irritation, risk of methemoglobinemia in young children, and possible complete suppression of flare responses in some cases [14, 40, 41]. These limitations highlight the value of non-pharmacological approaches like VR, which can provide effective pain and anxiety reduction without interfering with test accuracy or requiring extended preparation time.

The mechanism of pain reduction with VR is currently the subject of research, with studies focusing on attention-based theories and potential gate control mechanisms. When individuals interact with the virtual world, the brain may have fewer attention resources to cope with pain, potentially resulting in reduced pain perception [42, 43]. A functional magnetic resonance imaging (fMRI) study showed increased activity in neuroanatomical pain matrix regions when individuals were exposed to painful stimuli [44]. Hoffman et al. demonstrated that the activity of the individual pain matrix decreased by more than 50% with fMRI when patients received VR interventions [43]. Additionally, Pourmand et al. reported that VR was able to increase the body’s tolerance to pain by reducing patients’ negative emotions and shortening the time during which they perceived pain [45].

Previous studies have reported that VR significantly reduces fear associated with invasive procedures in children [19, 33, 46–48]. A randomized controlled trial by Gerçeker et al. showed that VR significantly reduced not only pain but also fear and anxiety levels in children during blood draws [33]. Erdoğan et al. reported that VR reduced procedural pain, fear, and anxiety in children aged 7–12 during intravenous insertion and blood draw [19]. Moreover, in another study, VR helped to reduce procedural pain, fear, and anxiety in children aged 10–21 during blood draw [46]. VR has been found to significantly reduce fear levels associated with needle-related procedures compared with traditional distraction methods in children in the emergency department [47]. Finally, a meta-analysis focusing on needle-related procedures also reported that VR significantly reduced fear [48].

Le May et al. showed that high levels of anxiety and fear can worsen the pain experienced by children and lead to a lower pain threshold [49]. A meta-analysis reported that VR was capable of reducing negative emotions by activating the advanced cognitive centers of the nervous system [50]. Additionally, the head-mounted display in VR can block the medical environment of pediatric cases and reduce patients’ environmental fears and protect them from negative visual and auditory stimuli, thus creating a pleasant diagnostic and treatment atmosphere [20].

### Strengths of the study

This research represents a parallel-group prospective randomized controlled single-blind study. The participants were assigned to groups using block randomization, including gender and age group variables. Previous studies have shown a positive relationship between procedural pain and fear, and interventions aimed at reducing procedural pain therefore also affect procedural fear [29]. For that reason, pain and fear associated with skin prick testing were evaluated together in our study. The pain and fear levels reported by children are particularly valuable in such assessments because it is they who directly experience the pain and fear. However, self-report levels can vary depending on the child’s age, understanding, and perception levels. Pain and fear were therefore evaluated by three different assessors in this study, the child, a volunteer parent, and an experienced nurse, in order to minimize measurement errors and researcher bias.

### Limitations

Since the researchers themselves randomized the participants into groups, the study was not double-blind. The limited sample size and the restriction to a specific age group limit the generalizability of the results. Additionally, variables such as the type and duration of the VR content used in the study may also affect the magnitude of the effect. Additionally, while we carefully selected open-design VR goggles and adjusted straps individually, complete facial expression assessment remained challenging during VR procedures. This limitation was mitigated through our comprehensive evaluation strategy combining real-time behavioral observations with immediate post-procedure self-assessments, where children’s own reports served as the primary indicators of their pain and anxiety experience.

### Recommendations for future research

Although VR poses a number of challenges, such as cost and complexity, requiring additional personnel to manage it, and necessitating hygiene measures in hospital settings, future expectations for VR in managing pain and fear associated with invasive procedures are high. However, providing age-appropriate content and interfaces requires careful evaluation and standardization. As technology advances, personalized VR experiences should become more accessible and adaptable to individual patient needs. The ability of VR to simulate real-life scenarios offers a new means of preparing children for upcoming medical procedures, potentially reducing procedure-related pain and fear. Further studies with larger and more diverse samples and including a comparison of different types of VR content and aimed at overcoming potential barriers to technology access are now needed.

## Conclusions

In this first such study in the literature, VR emerged as effective in reducing pain and fear associated with skin prick testing in children. Considering its effectiveness on pain and fear associated with skin prick testing in children, VR appears to constitute a useful modality in other challenging diagnostic and treatment procedures due to its unique abilities to distract and soothe. A delicate balance between technology and traditional care must be established in order to achieve the most beneficial outcomes for children. In conclusion, while VR opens an exciting new door for the procedures in pediatric allergy clinics, it is also time to use it as a game-changer for a game-like skin test experience in this “Brave New World.”

## Relevance to clinical practice

Virtual reality (VR) can be a valuable tool in pediatric allergy clinics by reducing pain and fear associated with skin prick testing. By providing an engaging and immersive experience, VR helps children feel more comfortable and cooperative during procedures, potentially leading to improved procedural success rates. Additionally, reducing procedural distress may enhance children’s and parents’ willingness to attend follow-up appointments and adhere to allergy management plans. This, in turn, could contribute to better long-term clinical outcomes and improved quality of care in pediatric allergy practice.

## Data Availability

No datasets were generated or analysed during the current study.

## Abbreviations

CFS: Children’s Fear Scale
fMRI: Functional magnetic resonance imaging
VR: Virtual reality
WB-FACES®: Wong-Baker FACES® Pain Scale

## Declarations

### Statistics

The statistics were checked prior to submission by an expert statistician (Imran Kurt Omurlu; ikurtomurlu@gmail.com).
